# Mobile Phone App–Based Pulmonary Rehabilitation for Chemotherapy-Treated Patients With Advanced Lung Cancer: Pilot Study

**DOI:** 10.2196/11094

**Published:** 2019-02-04

**Authors:** Sojung Park, Ji Youn Kim, Jae Cheol Lee, Hyeong Ryul Kim, Seungjae Song, Hee Kwon, Wonjun Ji, Chang Min Choi

**Affiliations:** 1 Department of Pulmonary and Critical Care Medicine, Asan Medical Center University of Ulsan College of Medicine Seoul Republic of Korea; 2 Department of Outpatient Nursing, Asan Medical Center Seoul Republic of Korea; 3 Department of Oncology, Asan Medical Center University of Ulsan College of Medicine Seoul Republic of Korea; 4 Department of Thoracic and Cardiovascular Surgery, Asan Medical Center University of Ulsan College of Medicine Seoul Republic of Korea; 5 Life Semantics Corp Seoul Republic of Korea

**Keywords:** chemotherapy, physical fitness, lung cancer, rehabilitation, quality of life

## Abstract

**Background:**

Advanced lung cancer patients often have chronic lung disease with reduced exercise capacities and various symptoms leading to altered quality of life (QoL). No studies have assessed pulmonary rehabilitation (PR) employing a mobile app and an Internet of Things device in advanced lung cancer patients undergoing chemotherapy.

**Objective:**

This study aimed to determine the feasibility and efficacy of smartphone app–based PR on exercise capacity, symptom management, and QoL in patients with advanced lung cancer undergoing chemotherapy.

**Methods:**

A total of 100 patients were recruited in a prospective, single-arm intervention study using a smartphone app–based PR program for 12 weeks. Exercise capacity (6-min walking distance, 6MWD), QoL, symptom scale scores, and distress indexes were investigated.

**Results:**

A total of 90 patients completed the PR program. The most common cause of drop out was hospitalization because of cancer progression. After PR, there was significant improvement in the 6MWD; 380.1 m (SD 74.1) at baseline, 429.1 m (SD 58.6) at 6 weeks (*P*<.001), and 448.1 m (SD 50.0) at 12 weeks (*P*<.001). However, the dyspnea scale score showed no significant improvement in the patients overall, but there was a trend for improvement in those with a stable tumor response (*P*=.07). Role (*P*=.02), emotional (*P*<.001), and social functioning (*P*=.002) scale scores showed significant improvement after PR. Symptom scale scores for fatigue (*P*<.001), anorexia (*P*=.047), and diarrhea (*P*=.01) also showed significant improvement. There was significant improvement in depression (*P*=.048) and anxiety (*P*=.01), whereas there was no significant change in QoL (*P*=.06) and severity of pain (*P*=.24).

**Conclusions:**

Smartphone app–based PR represents an effective and feasible program to improve exercise capacity and to manage symptoms and distress in patients with advanced lung cancer who are undergoing chemotherapy.

## Introduction

### Background

Lung cancer is the leading cause of death worldwide. A large number of patients are diagnosed with lung cancer in advanced stage [1]. Although the median overall survival of patients with metastatic lung cancer remains poor, advances in cancer-targeting agents and immune checkpoint inhibitors have extended life expectancy [2-6]. Patients with lung cancer often have comorbidities, such as chronic obstructive pulmonary disease (COPD) and interstitial lung disease (ILD), associated with decreased lung function and increased respiratory symptoms [7]. In addition, the adverse effects of chemotherapy and various cancer symptoms alter quality of life (QoL) and reduce the physical activity and exercise capacity of patients undergoing chemotherapy [8].

Exercise capacity is associated with lung cancer prognosis [9]. Pulmonary rehabilitation (PR) has emerged as a cost-effective intervention for managing chronic lung disease. PR improves the 6-min walking distance (6MWD), QoL, and respiratory symptoms in patients with COPD and ILD [10-13]. However, there are very limited data to demonstrate the feasibility and efficacy of PR in advanced lung cancer patients [14-17]. Currently, a large number of patients with lung cancer receive chemotherapy as outpatients. Moreover, many live in areas where an attending physician is not readily accessible. Therefore, home-based PR and symptom management is essential for patients with advanced lung cancer. The use of mobile health care may assist in overcoming these barriers, improving educational levels, facilitate frequent assessments, and allow more objective data collection [18,19].

### Objectives

We investigated the feasibility and efficacy of a smartphone app–based PR and educational program on exercise capacity, symptom management, and QoL in patients with advanced non–small cell lung cancer (NSCLC) undergoing chemotherapy.

## Methods

### Participant Identification

Consecutive patients with histologically diagnosed advanced NSCLC were identified from September to November 2016 in Asan Medical Center, Seoul, South Korea. All patients were eligible to receive palliative chemotherapy or adjuvant chemotherapy. The inclusion criteria were as follows: patients aged 20 to 75 years with (1) NSCLC stage Ⅱ-Ⅳ, (2) Eastern Cooperative Oncology Group-Performance Status (ECOG-PS) 0-2, and (3) having an android smartphone and the ability to handle the app. Patients were excluded if they (1) had concurrent malignancies other than lung cancer, (2) had received prior PR or education on lifestyle modification, (3) had symptomatic heart disease, (4) had severe cognitive impairment, and (5) were not willing to make regular visits.

Written informed consent was obtained from all patients in accordance with the Declaration of Helsinki. This study was approved by the institutional review board of Asan Medical Center (proposal identification number 2016-0445).

### App for Mobile Health Care

A comprehensive mobile health care app, the Smart Aftercare app, was newly developed for this study. The English version of the brochure introducing the function of the app is found in Multimedia Appendix 1. Patients were provided with the Smart Aftercare app, an Internet of Things (IoT) wearable device (URBAN S, Partron Co, Seoul, Korea), which interlocked with the app, a portable pulse oximeter, thermometer, scale, and resistance bands for physical therapy.

The app engineers (Life Semantics Co, Seoul, Korea) developed the app with support from medical personnel. The app had a to-do list, individual health information, and an in-app chat service. The to-do list provided an alarm notification for daily tasks related to taking medication, performing rehabilitation exercise, and visiting the clinic on schedule. The app provided patients’ laboratory results, 1 to 3 key computed tomography images, and information on the efficacy and adverse events of the chemotherapy regimen patients were receiving, as well as general lung cancer information. Through the in-app chat service, a clinical nurse specialist also provided a counseling service.

### Pulmonary Rehabilitation Program

This study consisted of a 12-week rehabilitation program. The Smart Aftercare app provided an animation video on 10-min stretching exercises, 30-min aerobic exercises, 30-min muscle strengthening exercises, and 5-min finishing (stretching) exercises. Patients were instructed to run the IoT device and app during their exercise. The IoT device, which facilitates accelerometer-based activity monitoring, recorded patients’ activity, including the number of steps taken and walking distance, as well as heart rate (HR). This information was transmitted to the IoT platform, developed for this study, allowing data sharing with the attending physician to check patients’ condition in real time, objectively. To promote regular exercise, push notifications were sent to patients when the app had not been used for a period.

All patients performed the 6-min walk test (6MWT) every time they visited the clinic. The attending physician prescribed individualized exercise duration and intensity, which were adjusted after every clinic visit according to the results of the 6MWT. Walking, bicycle ergometer, and treadmill use were recommended. Exercise was prescribed as follows: once a day for 30 to 60 min at least three days a week, a walking distance target of 60% to 80% of the 6MWT, with an HR target of 70% of HR reserve plus resting HR (target HR=70% x [HRmax-HRrest] + HRrest). HRmax stands for maximum HR and HRrest stands for resting HR. Patients were instructed to quit exercising if their oxygen saturation fell below 88% or they could not talk with others because of dyspnea.

The muscle strengthening exercise program comprised strengthening of all major muscle groups in the limbs and trunk. Patients were instructed to perform strengthening exercises once a day for 30 min. Patients’ muscle strength was assessed by arm abduction test, 10 times. Various resistance bands with different intensities were provided according to patients’ muscle strength. The app and IoT device checked whether the patient completed daily exercise tasks and gave feedback on the amount of activity and calories expended in a day.

### Symptom Management

The Smart Aftercare app provided an animation video on pain control, nutritional support, and symptom management. Furthermore, algorithms for the management of pain and adverse skin reactions, a frequently asked questions service, were also provided. Patients were instructed to record daily body weight and temperature on the app.

### Data Collection

Demographic information was investigated at baseline, and the relevant medical data were collected from medical records. Patients visited the clinic every 4 or 6 weeks, depending on their schedule of chemotherapy (Table 1). At visits, they completed the questionnaires for QoL, symptom (the European Organization for Research and Treatment of Cancer Quality of Life Questionnaire-C30, EORTC QLQ-C30), pain (numeric rating scale, NRS), and distress, including anxiety (Generalized Anxiety Disorder-7, GAD-7) and depression (Patient Health Questionnaire-9, PHQ-9). We also evaluated service satisfaction using a self-developed questionnaire (Multimedia Appendix 2). For patients who visited the clinic every 4 weeks, the mean value of each scale at 4 and 8 weeks was used for investigation at midterm assessment. Permission to use the EORTC QLQ-C30 was obtained, and the authorized Korean version of the questionnaire was freely downloadable from the internet. No permission was required for NRS, PHQ-9, or GAD-7.

### Unexpected Visits to the Emergency Department

As we performed a single-arm study using a mobile app, this study was limited in that it had no control group. Therefore, we retrospectively evaluated the number of unexpected visits to the emergency department (ED) comparing patients who participated in this study with those who did not. The number of visits to the ED is widely regarded as an indicator of the development of unexpected, uncontrolled symptoms. We expected that the mobile app could provide information regarding how to manage symptoms through an algorithm and in-app chat service, thereby reducing medical expenses and time. From September to November 2016, a total of 1091 patients underwent chemotherapy in Asan Medical Center. Of these, 100 patients participated in this study, whereas 991 did not. Their electrical medical records were retrospectively reviewed.

**Table 1:** Assessment schedule.

Schedule	Screening	4, 8, or 6 weeks (±1 week)^a^	12 weeks (±1 week)^a^
Informed consent	✓^b^	—^c^	—
Demographic characteristics	✓	—	—
Inclusion or exclusion criteria	✓	—	—
Vital signs and oxygen saturation	✓	✓	✓
Physical examination	✓	✓	✓
History taking	✓	—	—
Disease status evaluation^d^	✓	✓	✓
Satisfaction questionnaire for the Aftercare app	—	—	✓
6-min walk test and exercise prescription	✓	✓	✓
EORTC QLQ-C30^e^	✓	—	✓
Numeric pain rating scale	✓	✓	✓
GAD-7^f^	✓	✓	✓
PHQ-9^g^	✓	✓	✓
Assessment for adverse reaction of chemotherapy	—	✓	✓

^a^1 week before and after the visiting day was allowed.

^b^Data were obtained at the time marked with a check.

^c^Data not obtained at the time marked with an dash.

^d^Treatment response was assessed at least once in all patients during the study period depending on their schedule of chemotherapy.

^e^EORTC QLQ-C30: European Organization for Research and Treatment of Cancer Quality of Life Questionnaire-C30.

^f^GAD-7: Generalized Anxiety Disorder-7.

^g^PHQ-9: Patient Health Questionnaire-9.

### Statistical Analysis

Categorical variables were analyzed using Pearson chi-square test or Fisher exact test. Continuous variables were analyzed using a Student *t* test. A paired *t* test and Bonferroni correction were used for comparison of pre- and post-PR assessment. All tests of significance were two sided, and differences between groups were considered to be significant when the *P* value was <.05. All statistical analyses were performed with SPSS software version 22.0 (IBM Corp, Armonk, NY).

## Results

### Patients

A total of 100 patients were enrolled, and 90 patients completed the 12-week rehabilitation program. The baseline characteristics of the patients are presented in Table 2. The mean age of the patients was 55.1 years (SD 8.7); 46.0% (46/100) were males. The most common cause of drop out was hospitalization because of cancer progression (6/100, 6.0%), followed by transfer to other hospitals (2/100, 2.0%) and difficulty in handling the app (2/100, 2.0%). Patients who had completed the PR program had a significant higher baseline body mass index and better performance status than patients who had dropped out.

### Exercise Capacity

**Figure 1:**
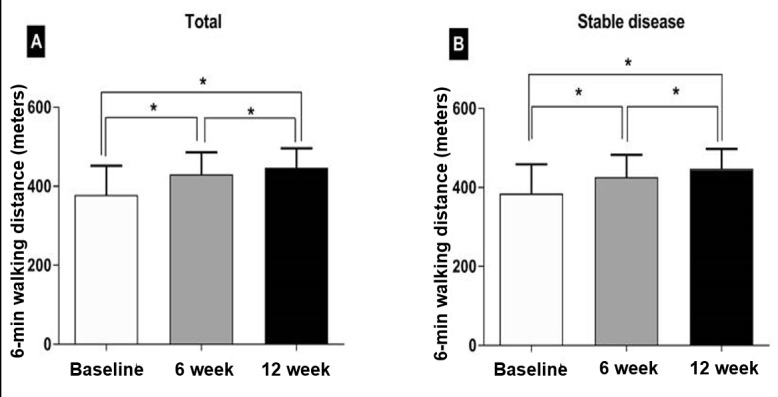
Exercise capacity. Six-minute walking distance improved significantly after pulmonary rehabilitation in the patients overall (A) and patients with stable tumor response (B).

The mean exercise number per week was 3.8 (SD 1.2) at 1 week, 4.2 (SD 1.1) at 6 weeks, and 4.1 (SD 1.2) at 12 weeks, satisfying the exercise prescription. A total of 85 patients completed all 6MWTs according to the schedule. In addition to 10 patients who dropped out, 5 patients refused to perform 6MWT because of general weakness, paraplegia, knee pain, and dizziness. There was significant difference in the baseline 6MWD according to baseline ECOG-PS. The mean distance was 416.8 m (SD 55.4) in patients with ECOG-PS 0, 369.8 m (SD 80.3) in those with ECOG-PS 1, and 305.7 m (SD 89.1) in those with ECOG-PS 2 (*P*=.04). After PR, the 6MWD had improved significantly: 380.1 m (SD 74.1) at baseline, 429.1 m (SD 58.6, *P*<.001) at 6 weeks, and 448.1 m (SD 50.0, *P*<.001) at 12 weeks (Figure 1). We investigated the exercise capacity depending on their treatment response. Consequently, patients with stable disease showed significantly improved 6MWD: 384.2 m (SD 74.6) at baseline, 426.1 m (SD 6.5, *P*<.001) at 6 weeks, and 447.4 m (SD 50.4, *P*<.001 at 12 weeks (Figure 1). However, exercise capacity remained unimproved in patients with progressive disease. The dyspnea scale, evaluated using the EORTC QLQ-C30, did not show any significant improvement in the patients overall, but patients with stable disease tended to improve.

### Quality of Life, Functional, and Symptom Scales

Of 90 patients who completed the study, 86 patients completed all questions in the questionnaire EORTC QLQ-C30. The EORTC QLQ-C30 consists of a global health status and QoL scale, functional scales, and symptom scales. Global health status and QoL tended to improve in patients overall, although not statistically significant (Table 3), whereas all functional scales, except the cognitive scale, improved significantly after PR. Symptom scales, in which a high score represents more severe symptoms, for fatigue, appetite loss, and diarrhea showed significant improvement in patients overall. In patients with stable disease, global health status and QoL scale did not improve significantly. Nevertheless, similar improvement in functional and symptom scales were observed in these patients as in the patients overall.

### Pain Control

**Figure 2:**
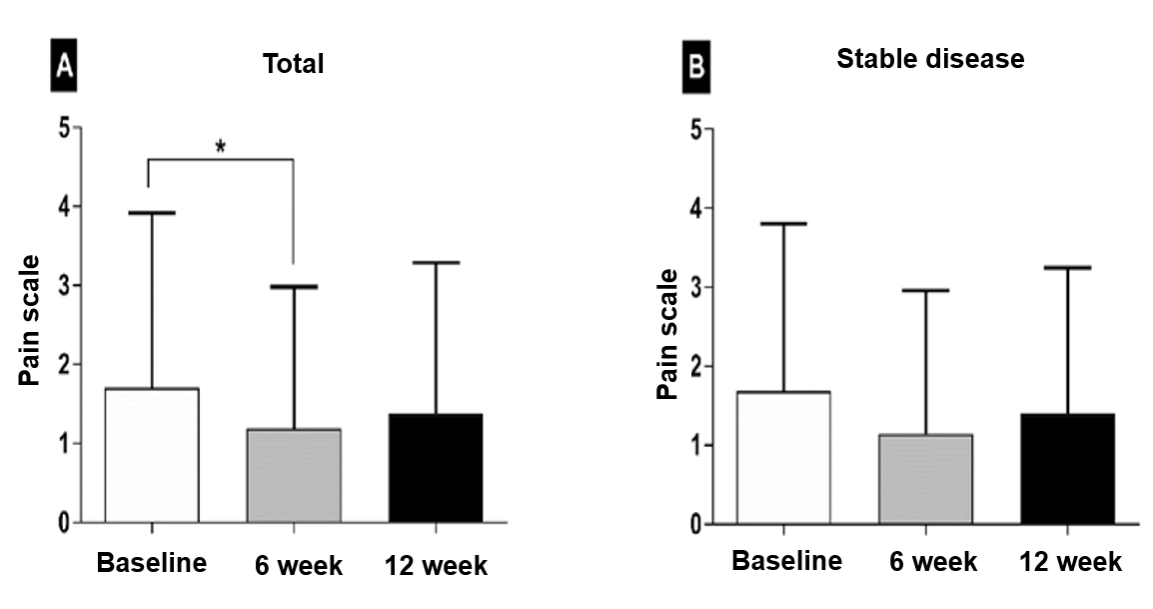
Pain scale. The pain severity, assessed by numeric rating scale, significantly decreased at 6 weeks but increased at 12 weeks (A). There was no significant improvement in patients with stable tumor response (B).

Overall, the pain severity significantly decreased at 6 weeks: 1.7 (SD 2.2) at baseline and 1.2 (SD 1.8, *P*=.02) at 6 weeks, but not at 12 weeks (mean 1.4, SD 1.9; *P*=.20; Figure 2). In patients with stable disease, the NRS score tended to improve at 6 weeks: 1.7 (SD 2.2) at baseline and 1.2 (SD 2.0, *P*=.06) at 6 weeks, but not at 12 weeks (mean 1.5, SD 1.9; *P*=.79).

### Distress Index

**Figure 3:**
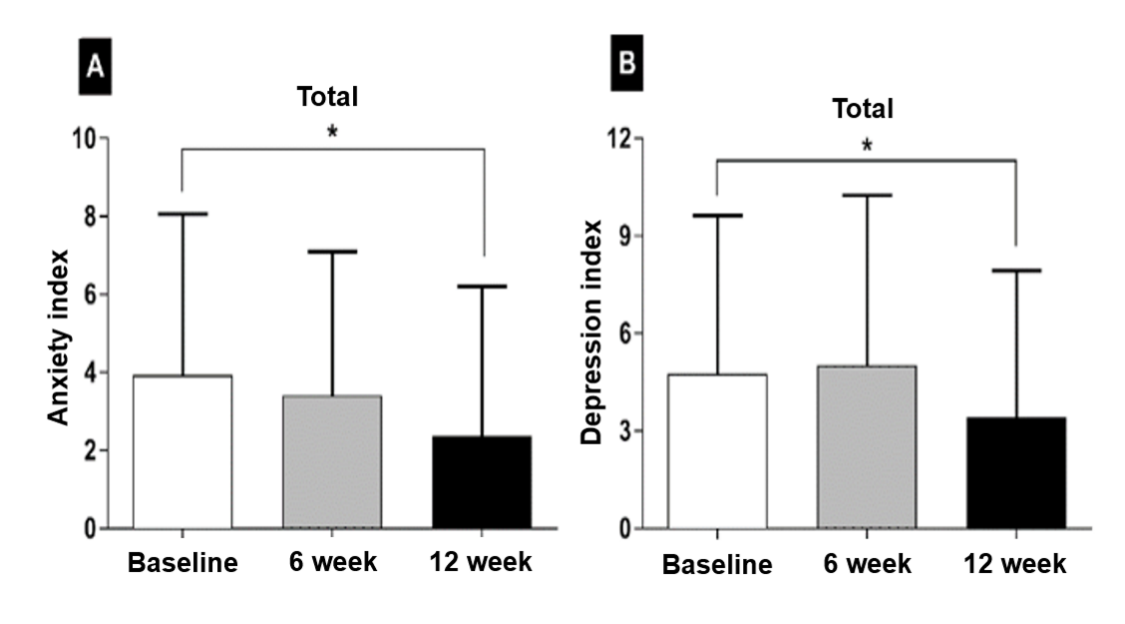
Distress indexes. Anxiety (A) and depression (B) significantly improved after pulmonary rehabilitation.

Overall, baseline distress indexes showed mild anxiety and depression (Figure 3). Low indexes represent less distress. Anxiety significantly improved at 12 weeks: 3.9 (SD 4.1; baseline), 3.4 (SD 3.7; 6 weeks *P*=.11), 2.4 (SD 3.8; 12 weeks, *P*<.001; Figure 3). Depression worsen at 6 weeks: 4.7 (SD 4.9; baseline), 5.0 (SD 5.2; 6 weeks, *P*=.44), but significantly improved at 12 weeks (mean 3.5, SD 4.5; *P*=.02; Figure 3). Only the depression index was associated with the treatment response (*P*=.04).

### Service Satisfaction

Of 90 patients who completed the PR program, 69 (69/90, 77%) patients reported that they were satisfied with the service and 79 (79/90, 88%) reported that they would recommend it to others. Neither age nor home region affected on service satisfaction. A total of 86 (86/90, 96%) patients reported that they were paying more attention to their health or disease status since using the app. In addition, all patients reported that the management algorithms for adverse events were helpful for controlling symptoms and determining when to visit the hospital. Patients who reported dissatisfaction with the service mostly cited difficulty in handling the app and frequent system error.

### Unexpected Visits to the Emergency Department

Of 991 patients who did not participate in this study, 302 (302/991, 30.5%) visited the ED. However, 15 (15/100, 15.0%) of 100 patients included in this study visited the ED during the same period; this indicated a significant reduction in frequency (*P*=.001). The baseline characteristics, such as sex and tumor stage, were not significantly different between 2 groups; baseline age was more in patients who did not participate in the study than in patients who did (62.9 years, SD 10.3 vs 55.2 years, SD 8.7; *P*=.03). However, there was no significant difference in age between patients who visited the ED and those who did not (62.6 years, SD 10.6 vs 63.0 years, SD 0.2; *P*=.71).

**Table 2:** Baseline characteristics of patients who participated in the mobile comprehensive rehabilitation program.

Baseline characteristics		Total (N=100)	Complete (n=90)	Interrupted (n=10)	*P* value
Age in years, mean (SD)		55.1 (8.7)	54.9 (8.8)	56.8 (7.7)	.53
**Sex, n (%)**					
	Male	46 (46.0)	41 (46)	5 (50)	>.99
BMI^a^ in kg/m^2^, mean (SD)		24.2 (3.5)	24.4 (3.5)	22.0 (1.8)	.04
**History of smoking, n (%)**					.59
	Current smoker	11 (11.0)	9 (10)	2 (20)	
	Ex-smoker	28 (28.0)	26 (29)	2 (20)	
	Never smoker	61 (61.0)	55 (61)	6 (60)	
**ECOG^b^, n (%)**					.02
	Zero	13 (13.0)	11 (12)	2 (20)	
	One	83 (83.0)	77 (86)	6 (60)	
	Two	4 (4.0)	2 (2)	2 (20)	
**Time of diagnosis of lung cancer, n (%)**					.82
	Within 1 year	56 (56.0)	49 (54)	7 (70)	
	Within 1-2 years	16 (16.0)	15 (17)	1 (10)	
	Within 2-3 years	16 (16.0)	15 (17)	1 (10)	
	>2 years	12 (12.0)	11 (12)	1 (10)	
**Stage, n (%)**					>.99
	II	5 (5.0)	5 (6)	0 (0)	
	III	0 (0.0)	0 (0)	0 (0)	
	IV	95 (95.0)	10 (100)	85 (94)	
**Lung function, mean (SD)**					
	FVC^c^ (%)	84.2 (17.7)	84.4 (17.3)	82.3 (23.6)	.79
	FEV_1_^d^ (%)	80.7 (19.2)	80.9 (19.3)	79.2 (19.5)	.84
	Diffusing lung capacity (%)	82.2 (15.1)	82.8 (15.0)	73.3 (15.2)	.22
**Line of chemotherapy, n (%)**					.76
	First	73 (73.0)	66 (73)	7 (70)	
	Second	12 (12.0)	10 (11)	2 (20)	
	Third and more	10 (10.0)	9 (10)	1 (10)	
	Adjuvant	5 (5.0)	5 (6)	0	
**Histology, n (%)**					.70
	Adenocarcinoma	94 (94.0)	84 (93)	10 (100)	
	Squamous cell carcinoma	4 (4.0)	4 (4)	0	
	Others	2 (2.0)	2 (2)	0	
**Regimen of chemotherapy, n (%)**					.78
	Tyrosine kinase inhibitor	40 (40.0)	36 (40)	4 (40)	
	Platinum-based chemotherapy	38 (38.0)	35 (39)	3 (30)	
	Others	22 (22.0)	19 (21)	3 (30)	
**Response rate^e^, n (%)**					.001
	Complete response	0 (0.0)	0 (0)	0 (0)	
	Partial response	15 (15.0)	14 (16)	1 (10)	
	Stable disease	69 (69.0)	66 (73)	3 (30)	
	Progressive disease	16 (16.0)	10 (11)	6 (60)	

^a^BMI: body mass index.

^b^ECOG: Eastern Cooperative Oncology Group.

^c^FVC: forced vital capacity.

^d^FEV_1_: forced expiratory volume in 1 second.

^e^Disease response was evaluated using response evaluation criteria in solid tumors criteria in patients with stage IV lung cancer. We considered patients with stage II lung cancer to have stable disease status.

**Table 3:** Quality of life, functional, and symptom scale.

EORTC QLQ-C30^a^		Total (n=86)			Stable disease (n=65)			Progressive disease (n=7)		
		Baseline, mean (SD)	12 week, mean (SD)	*P* value	Baseline, mean (SD)	12 week, mean (SD)	*P* value	Baseline, mean (SD)	12 week, mean (SD)	*P* value
Global health status or QoL^b^ scale		64.1 (24.7)	69.3 (21.2)	.06	66.3 (22.0)	70.5 (19.7)	.15	53.6 (35.0)	67.9 (26.5)	.24
**Functional scales**										
	Physical functioning	78.2 (14.3)	81.1 (15.7)	.06	77.6 (14.5)	81.2 (15.5)	.06	79.0 (18.2)	77.1 (20.7)	.63
	Role functioning	75.0 (22.8)	81.4 (23.3)	.02	75.6 (22.5)	82.1 (23.4)	.048	73.8 (30.2)	73.8 (33.1)	>.99
	Emotional functioning	73.7 (19.6)	83.7 (18.7)	<.001	74.9 (18.8)	82.9 (19.2)	.002	76.2 (21.2)	86.9 (19.8)	.33
	Cognitive functioning	81.4 (15.8)	83.9 (18.7)	.25	81.3 (15.5)	82.8 (20.2)	.54	88.1 (18.5)	85.7 (17.8)	.82
	Social functioning	74.6 (25.1)	82.8 (20.2)	.002	73.6 (25.5)	83.3 (20.2)	.001	73.8 (23.3)	83.3 (21.5)	.23
**Symptom scales**										
	Fatigue	35.7 (21.2)	27.1 (22.3)	<.001	35.0 (22.1)	26.8 (22.2)	.001	36.5 (24.6)	34.9 (29.0)	.86
	Nausea or vomiting	8.9 (15.9)	10.5 (14.7)	.45	8.2 (15.6)	10.8 (15.4)	.27	9.5 (13.1)	7.1 (8.9)	.36
	Pain	20.2 (20.9)	22.9 (23.6)	.33	19.0 (21.4)	25.4 (24.7)	.06	23.8 (16.3)	14.3 (15.0)	.23
	Dyspnea	26.7 (23.3)	25.2 (25.0)	.56	27.2 (22.7)	25.6 (23.4)	.07	19.0 (26.2)	38.1 (44.8)	.10
	Insomnia	26.0 (28.2)	21.3 (28.0)	.12	25.1 (29.5)	22.1 (27.2)	.39	19.0 (32.5)	28.6 (48.8)	.17
	Appetite loss	21.7 (25.4)	16.3 (21.5)	.047	21.5 (24.6)	14.9 (21.3)	.03	33.3 (38.5)	19.0 (26.2)	.20
	Constipation	15.5 (23.8)	17.1 (23.3)	.65	16.4 (25.8)	15.9 (22.9)	.90	14.3 (17.8)	23.8 (25.2)	.36
	Diarrhea	19.4 (26.8)	11.6 (19.6)	.01	21.0 (28.6)	10.8 (17.8)	.01	28.6 (23.0)	14.3 (26.2)	.08
Financial difficulties		24.8 (26.2)	21.3 (25.0)	.14	26.7 (27.8)	21.5 (25.3)	.049	28.6 (23.0)	19.0 (26.2)	.36

^a^EORTC QLQ-C30: European Organization for Research and Treatment of Cancer Quality of Life Questionnaire-C30.

^b^QoL: quality of life.

## Discussion

### Principal Findings

The 12-week Smart Aftercare PR program provided comprehensive management of common challenges of patients with advanced lung cancer. To the best of our knowledge, this is the first study that demonstrated the feasibility and efficacy of smartphone app–based PR for improving exercise capacity and symptoms in patients with advanced NSCLC during chemotherapy.

Because many lung cancer patients are elderly, there is some concern about difficulty in handling a smartphone app. However, only 2 patients ceased participation for this reason. The most important cause of drop out was cancer progression. A total of 16 patients suffered from cancer progression during the 12 weeks’ PR program, and 2 patients died. Patients with advanced cancer will unavoidably suffer symptomatic deterioration with disease progression. However, exercise capacity, functional status, various symptoms, and distress indexes significantly improved in patients with stable disease. The objective response rate to estimated glomerular filtration rate tyrosine kinase inhibitors exceeds 70%, and the median duration of response to an immune checkpoint inhibitor is 10 to 25 months [2,3,5,6]. Therefore, the role of PR in advanced NSCLC seems increasingly important.

Exercise capacity is associated with advanced lung cancer prognosis [9]. Multiple small trials have demonstrated the benefit of hospital-based and home-based PR in patients with advanced lung cancer [14-17]. However, no studies have assessed PR employing a mobile app. The 6MWD significantly improved after PR in patients overall as well as in patients with stable disease. Although the dyspnea scale was not significantly improved, it tended to improve in patients with stable disease. Baseline performance status was significantly associated with baseline 6MWD but not with changes in absolute distance. Rather, patients with ECOG-PS 1 showed a mean increase in walking distance of 17 m more as compared with patients with ECOG-PS 0. One patient with ECOG-PS 2 showed an increase in walking distance of 132 m after the 12-week PR. Therefore, PR should be recommended to patients regardless of their disease status or PS, if feasible.

Smartphone-based PR provided significant improvement in functional scales, symptomatic scales, and distress indexes. Of 90 patients who completed the PR program, 86 (86/90, 96%) patients reported that smartphone-based PR promoted behavioral changes and facilitated self-monitoring of symptoms. This might have affected functional scale improvement, especially emotional scales and distress indexes. In addition, Shallwani et al reported that 6MWT distance was the predictor of change in the mental component of QoL. Therefore, improvement of exercise capacity affected emotional scales and distress indexes [20]. Moreover, 73 (73/90, 81%) patients reported that they felt that they were in contact with their health care team. Indeed, 78 (78/100, 78.0%) patients enrolled in this study lived outside of Seoul. This approach might be useful for patients living in areas where an attending physician is not readily accessible. Patients who participated in this study had significantly fewer ED visits than patients who did not participate in the study. In addition, there were no adverse events related to the smartphone-based PR, such as condition deterioration because of immoderate exercise or medicine overdose.

Anxiety and depression were significantly improved at 12 weeks. Depression was associated with the treatment response. In subgroup analysis, patients who received first-line chemotherapy showed a subsequent reduction in depression index, with a significant reduction in score at 12 weeks (5.6 [SD 5.8] at baseline, 5.5 [SD 5.8] at 6 weeks, and 3.5 [SD 4.8] at 12 weeks; *P*=.04). Furthermore, patients who received second-line chemotherapy and beyond showed higher depression index at 6 weeks (4.1 [SD 3.0] at baseline vs 5.3 [SD 5.6] at 6 weeks; *P*=.24) but lower index at 12 weeks (3.4 [SD 3.9], *P*=.33). Therefore, disease status and duration of illness were important factors in the distress index.

QoL and severity of pain were not significantly improved through PR. Pain is a key factor affecting QoL in patients with lung cancer [21]. We provided various kinds of services to help reduce pain, but they were ineffective. Therefore, self- management of pain using a smartphone app is insufficient and frequent assessment and physical examination at a clinic is needed. In contrast, treatment response, an important factor affecting health-related QoL, showed no significant association with QoL in this study [22].

### Limitations

This study has several limitations. First, there was no control group. Uhm et al reported that mobile health management did not show significant superiority over a conventional program in terms of physical function in patients with breast cancer [23]. However, the lungs are strongly associated with dyspnea and exercise capacity, and the usefulness of home-based PR for chronic lung diseases and lung cancer has previously been reported [17,24,25]. Further randomized controlled studies are needed to prove the superiority of the smartphone app–based PR over conventional education. Second, a small number of patients with heterogeneous disease status were included. In addition, the influence of the different chemotherapy regimens, which are associated with different adverse reactions that influence symptoms and QoL, was not considered. Third, monitoring of the amount and intensity of exercise relied on the IoT device only. If the IoT device had a systemic error, all data gathered on the IoT platform are unreliable. However, several studies have demonstrated the viability of a smartphone for step counting or gait analysis, and thus, they can be used to automate the 6MWT [26-29].

### Conclusions

In conclusion, 12 weeks of comprehensive smartphone app–based individualized PR seems to be an effective and feasible approach for improving exercise capacity, symptom management, and distress in patients with advanced NSCLC undergoing systemic chemotherapy.

## Appendix

Multimedia Appendix 1 The English version of the brochure introducing the function of the Aftercare app.

Multimedia Appendix 2 Questionnaire for service quality and satisfaction.

## Abbreviations

6MWD: 6-min walking distance
6MWT: 6-min walk test
COPD: chronic obstructive lung disease
ECOG-PS: Eastern Cooperative Oncology Group-Performance Status
ED: emergency department
EORTC QLQ-C30: European Organization for Research and Treatment of Cancer Quality of Life Questionnaire-C30
FEV_1_: forced expiratory volume in 1 second
FVC: forced vital capacity
GAD-7: Generalized Anxiety Disorder-7
HR: heart rate
ILD: interstitial lung disease
IoT: Internet of Things
NRS: numeric rating scale
NSCLC: non–small cell lung cancer
PHQ-9: Patient Health Questionnaire-9
PR: pulmonary rehabilitation
QoL: quality of life
