# Supplementary figures and images for: Mobile Phone App–Based Pulmonary Rehabilitation for Chemotherapy-Treated Patients With Advanced Lung Cancer: Pilot Study
Source: JMIR Mhealth Uhealth. 2019 Feb 4;7(2):e11094. doi: 10.2196/11094 (PMC6378551; doi:10.2196/11094)

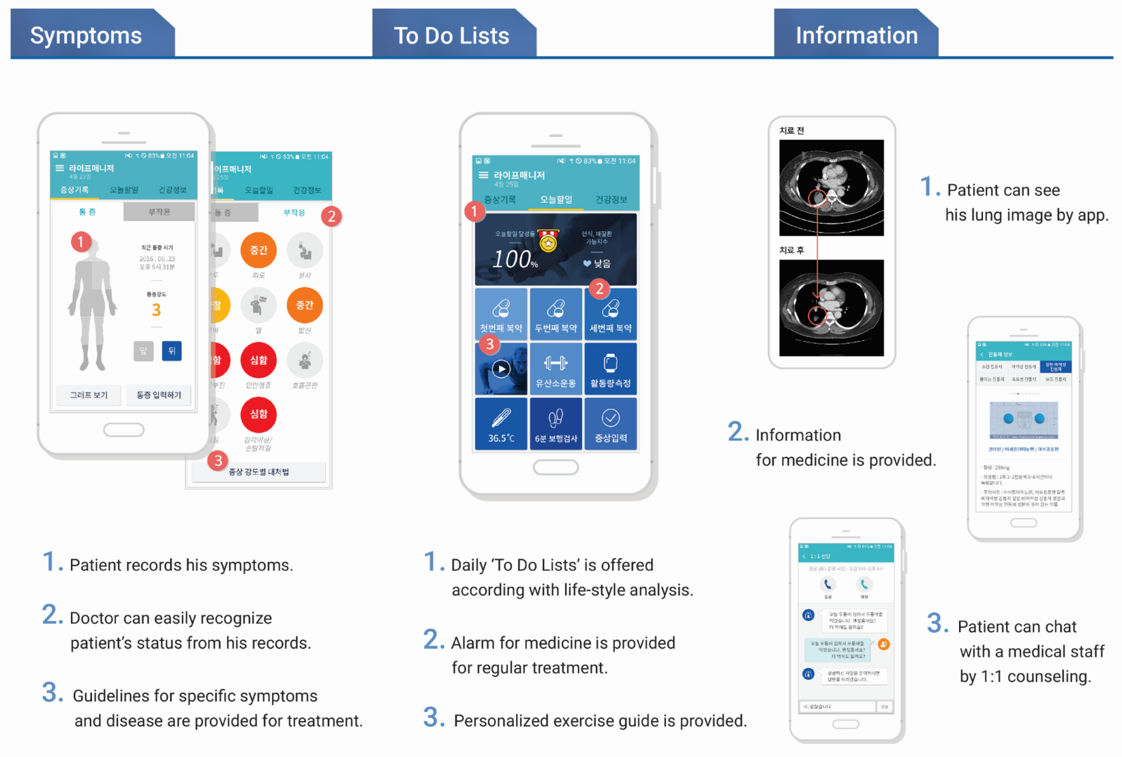

Supplement: Multimedia Appendix 1 [file mhealth_v7i2e11094_app1.png]
