# Supplementary material for: Mobile Phone App–Based Pulmonary Rehabilitation for Chemotherapy-Treated Patients With Advanced Lung Cancer: Pilot Study
Source: JMIR Mhealth Uhealth. 2019 Feb 4;7(2):e11094. doi: 10.2196/11094 (PMC6378551; doi:10.2196/11094)
Supplement: Multimedia Appendix 2 [file mhealth_v7i2e11094_app2.pdf]

Patient Initials				Subject Number								2

Date	2	0			-			-		

Service satisfaction survey

Ⅰ. Satisfaction in Smart Aftercare service

1. The following is a survey on the degree of satisfaction and future use of Smart Aftercare service.

1) How satisfied are you with the Smart Aftercare Service?	Very satisfied	Satisfied	Neutral	Dissatisfied	Not at all

2) If answering “Dissatisfied” or “Not at all” in the above question, what is the reason? (multiple responses are allowed)

① Not helpful for general healthcare
② Difficulty in handling application
③ Method too cumbersome
④ Does not provide immediate medical feedback or practical service
⑤ Less than adequate care as compared to conventional care
⑥ Affects household economy negatively (hospital expenses, transit expenses)
⑦ Causes incidental expenses (smartphone fee, internet fee)
⑧ Frequent change to clinic visit schedule by medical doctor
⑨ Too simple a treatment
⑩ Fear of loss of privacy
⑪ Systemic error (application error or IoT device error)
⑫ Miscellaneous ( )

3) Are you satisfied with the information taken from the Smart Aftercare Service?	Very satisfied	Satisfied	Neutral	Dissatisfied	Not at all

4) Are you willing to use the Smart Aftercare service after research completion?	Highly	Probably	Neutral	Probably not	Not at all

5) If answering "Highly" or "Probably" in the above question, what is the reason? (multiple responses are allowed)

① Good for general healthcare
② Convenient to use, regardless of time and place
③ Gives immediate medical feedback
④ Sufficient consultation from medical provider
⑤ Makes high-quality medical service available
⑥ Good for household economy
⑦ High-tech and professional service
⑧ Miscellaneous (   )

6) Are you willing to recommend Smart Aftercare service to other patients?	Highly	Probably	Neutral	Probably not	Not at all

7) If answering "Highly" or "Probably" in the above question, what is the reason? (multiple responses are allowed)

① Good for general healthcare
② Convenient to use, regardless of time and place
③ Gives immediate medical feedback
④ Sufficient consultation from medical provider
⑤ Makes high-quality medical service available
⑥ Good for household economy
⑦ High-tech and professional service
⑧ Miscellaneous (   )

2. The following is the survey on the device and program used for the Smart Aftercare service.

1) How satisfied are you with the quality of communication with the medical team provided by the Smart Aftercare Service?	Very satisfied	Satisfied	Neutral	Dissatisfied	Not at all

2) Was it easy to use the devices associated with the Smart Aftercare Service?	Very easy	Easy	Neutral	Not easy	Not at all

3) Were vital signs, assessed by the provided devices, correctly transmitted? Was it easy to use devices associated with the Smart Aftercare Service?	Excellent	Good	Neutral	Bad	Not at all

4) Was the network used for the service fast and stable?	Excellent	Good	Neutral	Bad	Not at all

5) Was the quality of the provided devices* appropriate for the service? * Device: IoT wearable device, thermometer, scale, etc.	Excellent	Good	Neutral	Bad	Not at all

6) Was the quality of the application and program appropriate for the service?	Excellent	Good	Neutral	Bad	Not at all

3. The following is the survey on errors of the device and program used for the Smart Aftercare service.

1) Have you ever experienced the trouble because of error during the Smart Aftercare service?

① Yes ② No

2) If answering 'Yes' in the above question, what was the cause of the error? (multiple responses are allowed)

① Device error ② Network error ③ Program error

④ Unskilled operator

⑤ Miscellaneous ( )

4. The following is the survey on the utility of the Smart Aftercare service.

1) Are you managing your health status more continuously than before you participated in the Smart Aftercare service?	Much more	Somewhat more	Neutral	Not more	Not at all

2) Do you pay more attention to your health management now than before you participated in the Smart Aftercare service?	Much more	Somewhat more	Neutral	Not more	Not at all

3) Did the animation videos facilitate your understanding of the contents?	Much more	Somewhat more	Neutral	Not more	Not at all

4) Were the CT images provided by the Smart Aftercare service helpful for understanding your disease status?	Very satisfied	Satisfied	Neutral	Dissatisfied	Not at all

5) Were the management algorithms for adverse events helpful for controlling symptoms and determining when to visit the hospital?	Very satisfied	Satisfied	Neutral	Dissatisfied	Not at all

6) Did you come to follow your doctor's advice better after using the Smart Aftercare service?	Much more	Somewhat more	Neutral	Not more	Not at all

7) Do you think your doctor referred to the information gathered by the Smart Aftercare service in making treatment decisions?	Excellent	Good	Neutral	Bad	Not at all

8) Are you willing to continue to visit the medical institution that provided the Smart Aftercare service?	Excellent	Good	Neutral	Bad	Not at all

9) Was the credibility of the medical institution that provided the Smart Aftercare service improved?	Highly improved	Improved	Neutral	Not improved	Not at all

10) Do you think that the Smart Aftercare service is a good approach to use for patients with severe disease (cancer and heart disease)?	Strongly agree	Somewhat agree	Neutral	Not agree	Strongly disagree

Patient Initials				Subject Number								2

5-1. Are you willing to use the Smart Aftercare service if it charges users for the service?
① Yes ② No

5-2. If answering 'yes' in the above question, how much are you willing to pay for a month?
